# Retraction: Prognostic significance of DAPK promoter methylation in lymphoma: A meta-analysis

**DOI:** 10.1371/journal.pone.0286175

**Published:** 2023-05-18

**Authors:** The PLOS ONE Editors

After this article was published, similarities were noted between this article and submissions by other research groups which call into question the validity and provenance of the reported results. In addition, during editorial follow-up, a number of data extraction and analysis errors were found and confirmed by the first author, meaning it is not clear the conclusions are supported.

In light of these issues, *PLOS ONE* cannot stand by the reliability of the reported research, and the *PLOS ONE* Editors retract this article [1].

HW agreed with the retraction. LYZ, ZBG, WBZ, LLZ, YNL, and XYP either did not respond or could not be reached.
